# A 5-year follow-up study on the efficacy and safety of ultrasound-guided laser ablation in elderly patients with papillary thyroid microcarcinoma: A retrospective, single-center study from China

**DOI:** 10.3389/fendo.2022.972589

**Published:** 2022-11-03

**Authors:** Zhang Juan, Liang Yongping, Xiaochen Han, Zhiwu Wang, Jingping Liu, Yongfeng Zhao, Wengang Liu, Ping Zhou

**Affiliations:** ^1^ Department of Breast Surgery, Tangshan People’s Hospital, North China University of Science and Technology, Tangshan, China; ^2^ Department of Ultrasound, Tangshan People’s Hospital, North China University of Science and Technology, Tangshan, China; ^3^ Department of Otolaryngology — Head and Neck, Tangshan People’s Hospital, North China University of Science and Technology, Tangshan, China; ^4^ Department of Medical Oncology, Tangshan People’s Hospital, North China University of Science and Technology, Tangshan, China; ^5^ Department of Ultrasound, Third Xiangya Hospital, Central South University, Changsha, China

**Keywords:** ultrasound, percutaneous thermal ablation, laser ablation, elderly patients, papillary thyroid microcarcinoma (PTMC)

## Abstract

**Objective:**

The aim of this study is to evaluate the long-term efficacy and safety of ultrasound-guided percutaneous laser ablation (PLA) for the treatment of elderly patients with papillary thyroid microcarcinoma (PTMC).

**Methods:**

From September 2015 to April 2017, 38 elderly patients with PTMC confirmed through fine-needle aspiration biopsy (FNAB) were treated with PLA. Before the treatment, the location and volume of the nodule together with the patients’ symptoms were evaluated. Twenty-four hours after the treatment, contrast-enhanced ultrasound (CEUS) was performed to evaluate the completeness of the ablation. To evaluate the volume of the ablation area and recurrence or metastasis, ultrasound examination was performed at 1, 3, 6, and 12 months after the treatment and every 6 months thereafter. FNAB was performed for any suspicious recurrence or metastasis lesions.

**Result:**

The ablation of all the 38 patients was all achieved completely as confirmed by CEUS. No obvious complications were found. The success rate of single ablation was 100%. The average follow-up time was 64.58 ± 5.29 months (60–78 months). By the time of the last follow-up, 31 (81.58%) ablation lesions disappeared completely and seven (18.42%) ablation lesions showed scar-like changes. The volume of nodules was 40.69 ± 16.45 mm^3^ before operation, which decreased to 0.22 ± 0.76 mm^3^ by the end of 42 months, and all nodules disappeared 4 years after ablation (*P* < 0.01). At 6, 12, 18, 24, 30, 36, and 42 months after ablation, the average volume reduction rates (VRRs) were 12.09%, 31.21%, 50.9%, 72.06%, 84.79%, 95.65%, and 100%, respectively. Of all the patients enrolled, one patient (2.6%) had local recurrence and was treated with PLA again. No regrowth of treated nodule or lymph node metastasis and distant metastases was detected.

**Conclusion:**

Ultrasound-guided PLA is effective and safe for the treatment of elderly patients with PTMC who are ineligible for surgery.

## Introduction

The incidence of papillary thyroid microcarcinoma (PTMC) is reported to have increased in recent years (1, 2). Among the newly diagnosed thyroid cancers, 50% cases are PTMC (3). Because of the low invasiveness of PTMC, the mortality rate is very low (4, 5). Moreover, the patients with PTMC rarely suffered from any symptoms associated with the disease itself even in those with lymphatic metastasis (6, 7). Until now, surgery is still the main treatment of thyroid cancer; however, the complications of the treatment (such as parathyroid and nerve injury) seriously affect patients (8–11). Some elderly patients are not suitable or unwilling to be treated with surgery. On the other hand, there were still several cases that progressed during surveillance (12, 13). Together with the anxiety associated with the disease during the surveillance, it is urgently needed to find a better treatment.

Ultrasound-guided PLA has developed rapidly in recent years and has been widely used in the treatment of liver, kidney, and lung tumors (14, 15). Our previous study has confirmed that PLA has high VRR and few complications for thyroid tumor (16). Here, we hypothesize that ultrasound-guided PLA can be effective and safe for the treatment of elderly patients with PTMC. Therefore, this trial was carried out to investigate the effect and safety of ultrasound-guided PLA in treating elderly patients with PTMC.

## Methods

### Patients

**Figure 1:**
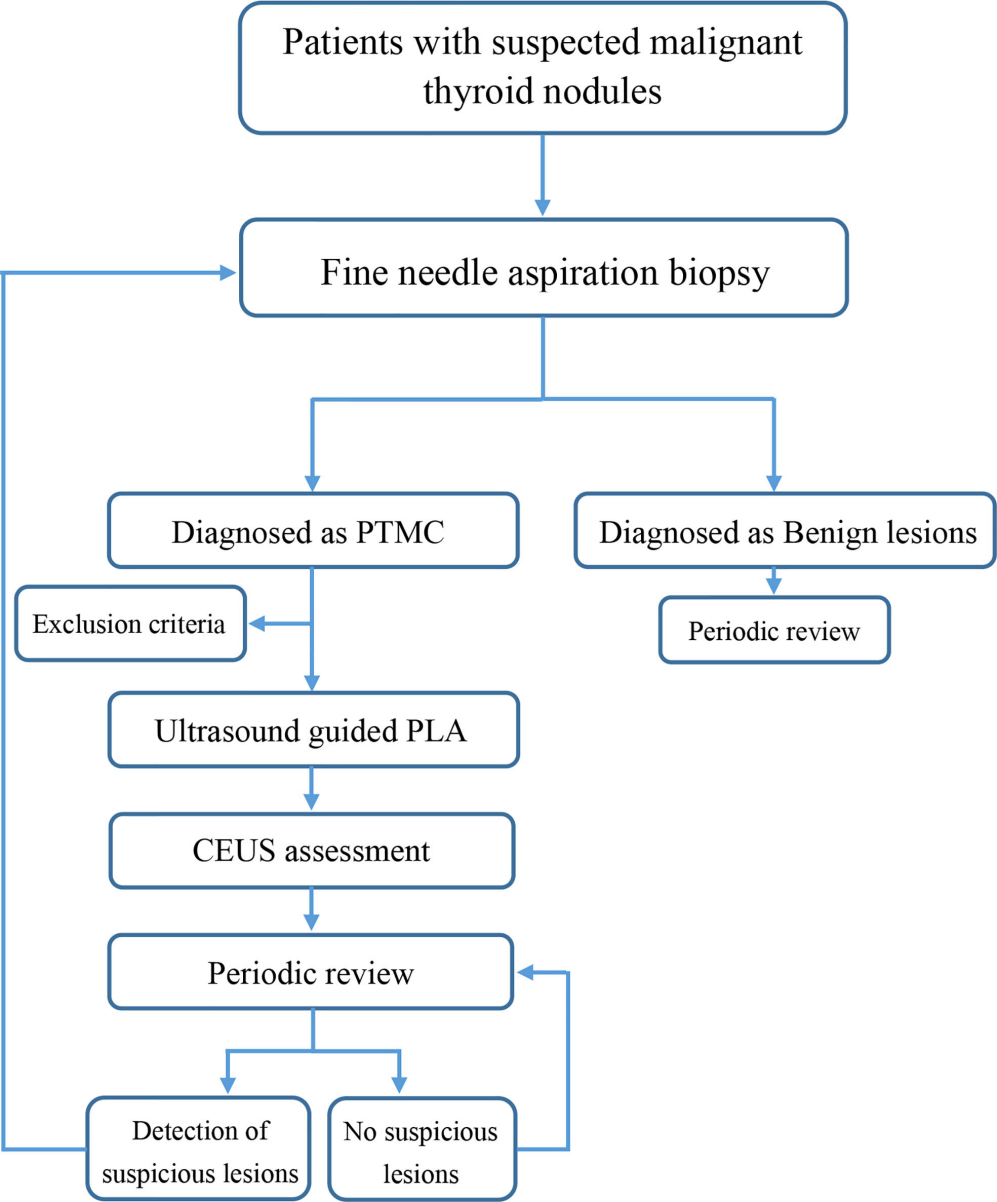
Study flowchart.

This retrospective trial was approved by the Ethics Committee, Third Xiangya Hospital, Central-South University, Changsha, China. From 1 September 2015 to 30 April 2017, elderly patients with pathologically confirmed PTMC were screened and treated with ultrasound-guided PLA in our department (**Figure 1**). The ablations were performed in accordance with approved guidelines and regulations, in a dedicated interventional operating room by two doctors (Ping Zhou, 20 years of service; Yong-feng Zhao, 11 years of service) who both had more than 10 years’ experience in performing PLA. The inclusion criteria were as follows (1): patients aged between 60 and 70 years who were diagnosed as papillary thyroid carcinoma; (2) single lesion with a maximum diameter less than 10 mm; (3) a minimum distance from the lesion to the thyroid capsule of ≥3 mm; (4) no tumor invasion to the extra-thyroid organs (trachea, common carotid artery, or esophagus); (5) patients refused surgery or could not tolerate surgery; and (6) patients who were anxious, which affected their normal life, or who are unwilling to undergo clinical observation. The exclusion criteria are as follows: (1) cytological diagnosis suggesting a more aggressive papillary cancer, or showing another type of thyroid malignancy such as medullary carcinoma; (2) clinically apparent multicentricity; (3) the lesions were close to the trachea, esophagus, or common carotid artery, and hydrodissection was difficult to establish; (4) family history of thyroid cancer; (5) adolescents or children with a history of neck radiation exposure; (6) abnormal blood routine, coagulation function, or major heart, lung, brain, and other diseases; (7) imaging examination showed suspicious neck and distant lymph node metastasis; and (8) unable to cooperate with the puncture. Each patient provided written informed consent before PLA after a full explanation of the purpose and nature of the procedure used.

### Equipment

The ultrasound system was a MyLab Twice color Doppler ultrasound system (Esaote, Italy) equipped with contrast-enhanced ultrasound imaging technology. High frequency linear array probe (6–12 MHz) were used for monitoring and pre-ablation assessment, ablation therapy, and follow-up.

PLA was conducted with an ultrasonic laser integrated system produced by Italian Esaote Medical and with an EchoLaser X4 laser (Nd : YAG laser) treatment system. The device comprised a 1,064-μm diode laser unit with a maximum of four laser sources, each with an individual energy emission setting and independent activation, a 0.3-mm-diameter optic fiber, a 21-gauge Chiba needle, and a foot pedal.

### Pre-ablation evaluations

Each patient underwent a cytologic examination by FNAB to confirm the diagnosis before ablation. The ultrasound examination was performed before ablation to identify and classify the target nodule and to characterize the anatomical relationship between the nodule and the important surrounding structures. The maximum diameter and two orthogonal diameters, the echoic characteristics, the internal blood flow distribution, and the ratio of the solid components of each nodule was obtained by an experienced sonographer. The nodule volume (V) was estimated by the ellipsoid formula V = π×a×b×c/6 (a is the maximum diameter; b and c are the two orthogonal diameters).

### Ablation procedure

**Figure 2:**
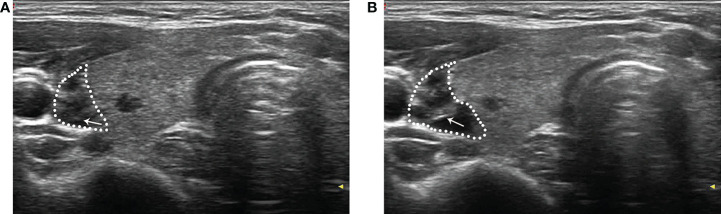
After local anesthesia, a mixture of 2% lidocaine and physiological saline was injected into the surrounding thyroid capsule by a 23-gauge needle (arrow in **A** and **B**), achieving a “hydrodissection” (dotted line in **A** and **B**) to protect the common carotid artery from thermal injury.

The patient was maintained at a supine position with the neck fully exposed. The target nodule location and its adjacent structures were evaluated with ultrasound, and the puncture route was predesigned. All procedures were performed under aseptic conditions and local anesthesia with 1% lidocaine. In order to protect the vital organs (trachea, recurrent laryngeal nerve, common carotid artery, and esophagus) from thermal injury, a bolus of 2% lidocaine and physiological saline solution (1:8 dilution) was carefully infused into the surrounding thyroid capsule to achieve a hydrodissection (17), the width of which was not less than 5 mm (**Figure 2**).

During the injection, short questions and answers were performed to monitor the status of the patient’s phonation. With appropriate anesthesia, as soon as the 21-gauge guide needle was percutaneously penetrated into the center of the target lesion under ultrasound guidance, the core needle was pulled out. A plane-cut optic fiber was inserted through the sheath of the 21-gauge needle to the same position. The ablation used an output power of 3 W. The specific energy may be changed according to the size of the nodule. When the lesion was completely covered by the hyperechoic area with a surrounding area of about 3 mm, the needle was removed while cauterizing the needle path with an output power of 3 W until the full needle was pulled out. The puncture point was disinfected and packed with asepsis gauze. The puncture point was pressed for 30 min to avoid bleeding. The ablation time and total energy were recorded, and hoarseness was evaluated immediately. The neck symptom scores were self-assessed by patients with a Visual Analogue Score (VAS) ranging from 0 (nil) to 10 (most severe).

### Post-ablation assessment

**Figure 3:**
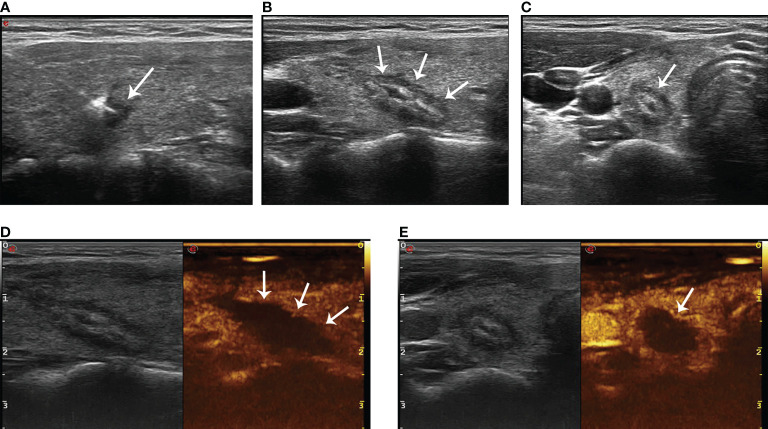
The hyperechoic area (arrow in **A**) around the pointed end of the optical fiber during PLA. The lesion (arrow in **B**, longitudinal plane; arrow in **C**, transverse plane) shows medium–high echogenicity in the ablation area and hypoecho in the surrounding area, with a clear boundary between the ablation area and normal tissue 24 h after ablation. There was no contrast agent perfusion in the ablation area (arrow in **D**, longitudinal plane; arrow in **E**, transverse plane) after PLA 24 h on CEUS.

Routine ultrasound and CEUS (SonoVue, Bracco, Milan, Italy) were performed 24 h after ablation to evaluate whether the ablation was complete (**Figure 3**). The ablative range, nodule echo, blood flow, and non-ablated portion were also recorded. Patients were not allowed to leave the hospital unless they have normal vital signs and no complications 24 h after ablation. The volume of the ablated nodules and the percentage change in volume after ablation (VRR, calculated as a percentage as follows: (initial volume - final volume) × 100/initial volume) were recorded at postoperative 1, 3, 6, and 12 months during the first year of follow-up and every 6 months thereafter. If suspicious lymph nodes or lesions in thyroid were found, ultrasound-guided FNAB was performed.

### Statistical analysis

Data analyses were performed using SPSS statistical software version 22.0 (SPSS, Chicago, IL). Categorical variables are expressed as frequencies, continuous variables as mean ± standard deviation. The self-paired t-test was used to compare the volume of thyroid nodules before and after ablation. A *P* value <0.05 was considered statistically significant.

## Results

### Patients

From September 2015 to April 2017, a total of 38 patients were screened in our department (**Table 1**). All the patients enrolled had only one lesion; thus, a total of 38 lesions were treated by ultrasound-guided PLA. All of the 38 patients enrolled had at least a 60-month follow-up. Among the 38 nodules treated, hydrodissection was performed in 34 (89.47%) cases.

**Table 1:** Baseline characteristics of patients with PTMC treated with PLA.

Item	Number
Subjects	38
Female, n (%)	26 (68.42)
Age, year (range)	62 ± 2.4 (60-67)
Diameter, mm (range)	5.04 ± 0.98 (3.5-6.8)
Volume, mm^3^ (range)	40.69 ± 16.45 (18.69-82.25)
Total energy, J (range)	1020.58 ± 109.39 (720-1200)
Ablative caloric per unit volume (J/mm^3^)	25.08 ± 6.65 (14.59-38.52)
Total ablation time, s (range)	340.19 ± 36.46 (240-400)
FU period, mo (n)	78 (2), 72 (5), 66 (13), 60 (18)

Values are reported as mean ± SD. FU, follow-up; mo, months; J, Joule.

### Follow-up evaluation

**Figure 4:**
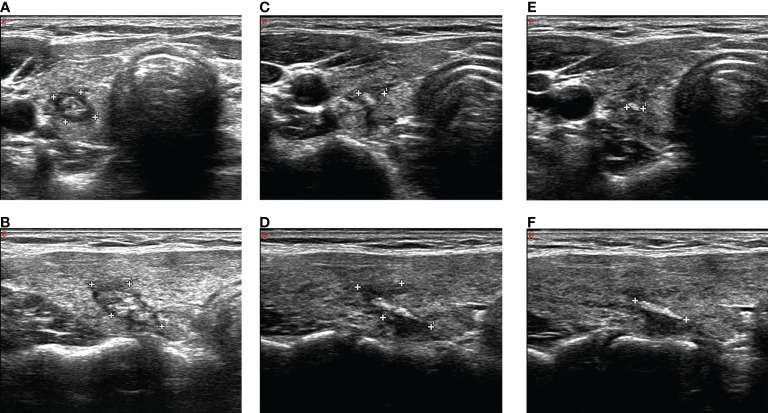
**A** The ablation lesion (caliper in A, transverse plane; caliper in **B**, longitudinal plane) shrank gradually after PLA 3 months. The ablation lesion (caliper in **C**, transverse plane; caliper in **D**, longitudinal plane) shrank further. The ablation lesion (caliper in **E**, transverse plane; caliper in **F**, longitudinal plane) shows scar-like changes after PLA 42 months.

Ultrasound 24 h after ablation showed medium–high echogenicity in the ablation area and hypoecho in the surrounding area, which has a clear boundary with the surrounding normal tissue. Color Doppler ultrasound showed no blood flow in the ablation area. CEUS showed that none of the patients enrolled had contrast agent perfusion in the ablation area. The original tumor area was completely covered and exceeded by the non-perfusion area, which suggested complete ablation. The success rate of single ablation was 100%. Within an average follow-up time of 64.58 ± 5.29 months (the longest follow-up time was 78 months), compared with the nodules before ablation, the volume of all ablation lesions was gradually reduced after ablation. By the time of 6, 12, 18, 24, 30, 36, 42, and 48 months after ablation, the average volumes of the ablated nodules were 35.77 ± 14.57, 27.99 ± 11.24, 19.98 ± 8.1, 11.37 ± 5.61, 6.19 ± 3.93, 1.77 ± 2.41, 0.22 ± 0.76, and 0 mm^3^, respectively (*P* < 0.01, all). The average VRR of ablation was 31.21%, 72.06%, 95.65%, and 100% by the time of 1, 2, 3, and 4 years after ablation, respectively. The mean maximum diameter and the mean volume of ablation were 5.04 ± 0.97 mm and 40.69 ± 16.45 mm^3^ before ablation, which decreased to 0.17 ± 0.61 mm and 0.22 ± 0.76 mm^3^ (*P* < 0.01) 42 months after ablation (**Table 2**), respectively. All lesions disappeared 4 years after ablation. Thirty-one (81.58%) ablation lesions disappeared completely, and seven (18.42%) ablation lesions left with scar-like changes (**Figure 4**).

**Table 2:** Outcomes of PTMC treated after ultrasound guided PLA.

Time	Max D, mm		Volume, mm^3^		VRR (%)	*P*
	Mean	scale	Mean	Scale	
BL	5.04 ± 0.97	3.5-6.8	40.69 ± 16.45	18.69-82.25	–	–
1M	10.69 ± 2.17	7.42-14.42	505.78 ± 207.52	212.35-1022.3	-1143	0.00
3M	9.03 ± 1.74	6.26-12.87	253.58 ± 102.54	116.49-517.56	-523.2	0.00
6M	4.82 ± 0.93	3.35-6.50	35.77 ± 14.57	16.5-73.1	12.09	0.00
12M	4.4 ± 0.85	3.05-5.93	27.99 ± 11.24	12.86-55.7	31.21	0.00
18M	3.68 ± 0.7	2.7-4.96	19.98 ± 8.1	9.22-40.27	50.9	0.00
24M	3.09 ± 1.08	0-4.42	11.37 ± 5.61	0-24.1	72.06	0.00
30M	2.29 ± 1.26	0-3.67	6.19 ± 3.93	0-13.23	84.79	0.00
36M	0.92 ± 1.3	0-2.95	1.77 ± 2.41	0-6.42	95.65	0.00
42M	0.17 ± 0.61	0-2.11	0.22 ± 0.76	0-2.88	99.46	0.00
4Y	0	0	0	0	100	0.00
5Y	0	0	0	0	100	0.00

BL, baseline; D, diameter; M, month; Y, year; VRR, volume reduction rate.

A 4-mm-sized tumor was found in the middle of the affected lobe near the anterior capsule close to the isthmus, which was confirmed as PTMC by FNAB in one patient’s non-ablative region of the thyroid gland by the time of 18 months after ablation. No obvious lymph node metastasis was found by imaging examination. After obtaining the patient’s consent, laser treatment was performed again. Since then, the patient was followed up for another 4 years already, and no local recurrence or lymph node metastasis was detected. Among the other 37 patients, no local recurrence or lymph node metastasis was detected. The recurrence-free survival rate within 5 years was 97.37%, and the metastasis-free survival rate was 100%.

### Safety assessment

The main complication of ablation was pain. There were 33 (86.84%) cases that obtained mild pain and five (13.16%) cases that obtained minor pain in the ablation area; the pain scores were 3.2 ± 1.1. Six (15.79%) patients complained that the pain radiated to the head, ears, shoulders, or teeth, especially when the ablated lesion was close to the anterior capsule. The pain disappeared within 2 weeks without any sequelae. No patient stopped the ablation because of pain. None of the patients had any complications such as trachea, esophagus, vascular injury, tissue swelling, or skin scald in the ablation area.

## Discussion

At present, ultrasound-guided PLA is a minimally invasive and common treatment for patients with thyroid nodule. A preliminary retrospective study showed that PLA is a clinically effective, well-tolerated, and safe treatment (18). However, there have been no previous studies on the application of PLA in the treatment of elderly patients with PTMC. In practice, some elderly patients were intolerant to take surgery due to poor physical condition, and for the reasons of cosmetic problems and psychological resistance, some elderly patients were unwilling to take surgery (19).

Ultrasound-guided PLA is confirmed to be a minimally invasive treatment for elderly patients with thyroid nodules (20). In addition, recent studies confirmed the safety and efficacy of PLA in the treatment of T1N0M0 thyroid cancer (21, 22). Few complications were encountered in PLA especially those adjacent to important organs and tissues. This may be because the laser fiber is more tenuous, which can be inserted through a fine needle, and the output energy of PLA is accurate and controllable, which means less trauma (23). However, the experience of PLA in the treatment for elderly patients with PTMC is still in the exploratory stage.

Zhou et al. (24) stated that the success rate of first-time ablation with laser was up to 99.9%. Yue et al. (25) and Pacella et al. (18) both reported that the success rate of first-time ablation with PLA was 100%. Consistent with previous studies, in our study, the success rate of first-time ablation in all patients was 100%. All of the 38 elderly patients of PTMC treated with PLA in our study were followed up over 5 years, with an average time of 64.58 ± 5.29 months. The optical fibers can be penetrated into the lesion by a 21-gauge Chiba needle, and the slim needle allows for more precise puncture of the nodule, which may be a reason for the high ablation success rate. In our study, both of the lesion and surrounding normal glands were covered by the gasification area, and total ablation energy was around 1,000 J. None of the thyroid and surrounding tissues did not show thermal damage after the ablation.

Zhang et al. (26) reported that the VRR of the tumor was close to 100% after ablation. In our study, 31 (81.58%) ablation lesions disappeared completely, and seven (18.42%) ablation lesions showed scar-like changes after 4 years. This may be because the local temperatures of laser ablation can rise to above 200°C, and the quantity of heat per unit of volume was about 25 J/mm^3^, which was nine times more heat than ablation of benign nodules (27). Therefore, the local tissue carbonization became more obvious, which was not conducive to full absorption. By the 5-year follow-up, one patient had local recurrence (the recurrence rate was 2.6%), who underwent ablation again and was followed up for another 4 years. No lymph node metastasis was observed. Dong et al. (28) reported that the recurrence rate of papillary thyroid carcinoma was up to 3.6% after surgery operation in the operated thyroid bed or contralateral residual thyroid tissue. Being concentrated, the transmission of the laser to the surrounding gland is less, together with the multifocal character of differentiated thyroid cancer and the highly invasive subtype of thyroid papillary carcinoma, which may lead to a local recurrence. Our study showed that the local curative effect was definite, and there was no lymph node metastasis, which proved that the curative effect of PLA was worthy of affirmation.

During the ablation process, patients complained of different degrees of pain. Most of the cases (33/38) got mild pain, and a few (5/38) patients got minor pain. Only one patient complained of severe pain half an hour after ablation and got relieved after ice compress. Zhao et al. (29) also reported that most patients have neck pain and burning during operation, which may be related to the location of nodules and the pain threshold of patients. There were no other obvious complications associated with PLA in this study. The reason may be that we adopted the “liquid isolation method” during the ablation of nodules, which isolate the lesion from adjacent important tissues and organs, and the output power of laser ablation was accurately controlled with low power (3 W).

Our study has several limitations. Firstly, ultrasonography is less sensitive to cervical lymph nodes, especially metastatic lymph nodes in area VI; thus, negative ultrasonography findings of the lymph node cannot rule out small metastasis. Secondly, the subtypes of PTMC of the patients in this study are not completely clear, which may be associated with different prognoses. Finally, this study is a single-center retrospective study with a relatively small sample size, which may not fully completely reflect the whole patient population.

## Conclusion

We demonstrated that ultrasound-guided PLA is effective and safe for the treatment of elderly patients with PTMC who are ineligible for surgery. The effectiveness and safety of this therapy need to be evaluated further in the future trial(s) with large sample size before it can be introduced for routine clinical use.

## Data availability statement

The original contributions presented in the study are included in the article/supplementary material. Further inquiries can be directed to the corresponding author.

## Ethics statement

The studies involving human participants were reviewed and approved by Ethics Committee, Third Xiangya Hospital, Central-South University, Changsha, China. The patients/participants provided their written informed consent to participate in this study.

## Author contributions

ZJ and LY-P have equal contributions to this article. ZJ: project administration and formal analysis. LY-P: conceptualization and writing—original draft. PZ: writing—review and editing. Y-FZ: resources. W-GL: supervision. X-CH: investigation. Z-WW: software. J-PL: validation. All authors contributed to the article and approved the submitted version.

## Funding

This work was supported by grants from the National Natural Science Foundation of China (No. 81871367), the Project of Hebei Provincial Health Commission (No. 20181231), the Natural Science Foundation of Hunan Province, China (No. 2021JJ31037), and the Project of Hunan Provincial Health Commission (No. 202209025123).

## Acknowledgments

The authors thank the participating patients and their families for their cooperation during the study.

## Conflict of interest

The authors declare that the research was conducted in the absence of any commercial or financial relationships that could be construed as a potential conflict of interest.

## Publisher’s note

All claims expressed in this article are solely those of the authors and do not necessarily represent those of their affiliated organizations, or those of the publisher, the editors and the reviewers. Any product that may be evaluated in this article, or claim that may be made by its manufacturer, is not guaranteed or endorsed by the publisher.

## Abbreviations

PTMC, papillary thyroid microcarcinoma; CEUS, contrast-enhanced ultrasound; FNAB, fine-needle aspiration biopsy; PLA, percutaneous laser ablation; VRR, volume reduction rates
